# Maternal and fetal risks of planned vaginal breech delivery vs planned caesarean section for term breech birth: A systematic review and meta-analysis

**DOI:** 10.7189/jogh.12.04055

**Published:** 2022-07-16

**Authors:** Francisco J Fernández-Carrasco, Delia Cristóbal-Cañadas, Juan Gómez-Salgado, Juana M Vázquez-Lara, Luciano Rodríguez-Díaz, Tesifón Parrón-Carreño

**Affiliations:** 1 Department of Gynaecology and Obstetrics, Punta de Europa Hospital, Cádiz, Spain; 2 Nursing and Physiotherapy Department, Faculty of Nursing, University of Cádiz, Algeciras, Spain; 3 Neonatal and Paediatric Intensive Care Unit, Torrecárdenas University Hospital, Almeria, Spain; 4 Department of Gynaecology and Obstetrics, Ceuta University Hospital, Midwifery Teaching Unit of Ceuta, University of Granada, Ceuta, Spain; 5 Safety and Health Postgraduate Programme, Espíritu Santo University, Guyaquil, Ecuador; 6 Department of Gynaecology and Obbstetrics, Ceuta University Hospital, Midwifery Teaching Unit of Ceuta, University of Granada, Ceuta, Spain; 7 School of Health Sciences, University of Almeria, Almeria, Spain; 8 Territorial Delegation of Equality, Health and Social Policies, Health Delegation of Almeria, Almeria, Spain

## Abstract

**Background:**

Breech presentation delivery approach is a controversial issue in obstetrics. How to cope with breech delivery (vaginal or C-section) has been discussed to find the safest in terms of morbidity. The aim of this study was to assess the risks of foetal and maternal mortality and perinatal morbidity associated with vaginal delivery against elective caesarean in breech presentations, as reported in observational studies.

**Methods:**

Studies assessing perinatal morbidity and mortality associated with breech presentations births. Cochrane, Medline, Scopus, Embase, Web of Science, and Cuiden databases were consulted. This protocol was registered in PROSPERO CRD42020197598. Selection criteria were: years between 2010 and 2020, in English language, and full-term gestation (37-42 weeks). The methodological quality of the eligible articles was assessed according to the Newcastle-Ottawa scale. Meta-analyses were performed to study each parameter related to neonatal mortality and maternal morbidity.

**Results:**

The meta-analysis included 94 285 births with breech presentation. The relative risk of perinatal mortality was 5.48 (95% confidence interval (CI) = 2.61-11.51) times higher in the vaginal delivery group, 4.12 (95% CI = 2.46-6.89) for birth trauma and 3.33 (95% CI = 1.95-5.67) for Apgar results. Maternal morbidity showed a relative risk 0.30 (95% CI = 0.13-0.67) times higher in the planned caesarean group.

**Conclusions:**

An increment in the risk of perinatal mortality, birth trauma, and Apgar lower than 7 was identified in planned vaginal delivery. However, the risk of severe maternal morbidity because of complications of a planned caesarean was slightly higher.

One of the most controversial topics in obstetrics in recent years has been the discussion about how to deal with breech delivery, whether vaginal or caesarean. Although caesarean is considered a safe way of treating breech delivery, it contributes to high rates of postpartum maternal morbidity in developed countries and it is known to cause significant complications such as anaemia, urinary tract infections, superficial or complete dehiscence of the operative wound, endometritis, inflammatory complications [1], muscle pain, headache, lack of sexual satisfaction after delivery, digestive problems, fever and infection, abnormal bleeding, and stress urinary incontinence [2].

However, in 2000, the authors of the Term Breech Trial Collaborative Group (TBT) [3] published a randomised multicentre collaborative study about how to deal with term breech delivery. They concluded that elective caesareans offered better results than vaginal deliveries in full-term foetuses with breech presentation, while maternal complications were similar between the two groups. So, according to this evidence, the practice of elective caesarean was fostered in such presentations [3]. Following this trend, the TBT recommendation was adopted by important organisations in many countries, opting for a scheduled caesarean before the end of gestation and this way preventing spontaneous breech vaginal delivery, and the attributed risks, from being triggered [4].

Subsequently, in 2006 the PREMODA multicentre study was published [5]. Based on this study, The American College of Obstetricians and Gynecologists changed their protocols that same year and concluded that vaginal delivery in breech presentation and single-term gestation was a reasonable option in properly selected pregnant women and experienced health workers [6].

Therefore, the TBT study [3] was called into question and some national associations [7] included the option of having a vaginal breech delivery in their childbirth care protocol for full-term breech presentation, allowing the free evolution of the delivery process, provided that there is specifically trained staff in the affected centre. This procedure is currently accepted [6].

Analysing the original TBT data [3], serious concerns were raised regarding the design of the study, methods, and conclusions. In a considerable number of cases, there was a lack of adherence to the inclusion criteria and there was great interinstitutional variation regarding the standards of care. Also, inadequate methods of foetal antepartum and intrapartum evaluation were used, and a large proportion of women were recruited during active delivery, in many cases, without assistance from a doctor with adequate experience [8].

Primary caesarean in the first pregnancy has been associated with neonatal and maternal adverse outcomes in subsequent delivery [9]. In this way, abandoning vaginal delivery with breech presentation and opting indiscriminately for a caesarean would mean denying women access to health care options [10].

The Cochrane review conducted by Hofmeyr et al., which focused on planned caesarean section for term breech delivery, concluded that it reduced perinatal and neonatal death as well as serious neonatal morbidity, at the expense of somewhat increased maternal morbidity compared with planned vaginal delivery. Authors suggested to consider mother's preference for vaginal birth and risks such as future pregnancy complications, and the option of external cephalic version [11].

The meta-analysis conducted by Berhan et al. [12] (1993-2014) aimed at assessing the risk of morbidity and perinatal mortality in breech, full-term and single-foetus deliveries. Results showed a higher relative risk in vaginal delivery for perinatal mortality, trauma at birth, and Apgar at the fifth minute of life.

The present meta-analysis sought to update scientific evidence with the latest studies published in the last 10 years (2010-2020), so the results would be a complementary update. The objective of this meta-analysis was to compare the risks of vaginal delivery with elective caesarean in breech presentations, in terms of neonatal mortality, perinatal trauma, Apgar, neonatal intensive care unit (ICU) admittance, and maternal morbidity, according to evidence published during the last 10 years.

## METHODS

### Study design

A systematic review of observational studies and meta-analysis was conducted. The Preferred Reporting Items for Systematic Reviews and Meta-Analyses (PRISMA) guidelines were followed [13,14].

### Sources

A systematic bibliographic search was carried out using the Cochrane, Medline, Scopus, Embase, Web of Science, and Cuiden databases. Extensive searches were performed on the reference lists of selected articles. Our search terms included: “breech”, “breech presentation”, “breech birth”, “breech delivery”. During the process, search terms were alternately combined using Boolean logic. The search was based on a clinically answerable question in PICO format, Population (pregnant women with single, full-term foetus, and breech presentation); Intervention (vaginal delivery risks); Comparison (caesarean delivery risks) and Outcomes (risk of neonatal mortality, perinatal trauma, Apgar test with low score, neonatal ICU admittance, and maternal morbidity). Following this structure, the different search strategies were designed. The detailed search strategies employed in each database are summarised in Table S1 in the **Online Supplementary Document**.

This revision protocol was registered in PROSPERO.

### Inclusion and selection criteria

For this study, the default inclusion criteria were:

1. Observational studies of cohorts were included; reviews, brief reports, guidelines, and comments were excluded.
2. Studies that assessed perinatal mortality and morbidity in relation to the type of delivery with breech presentation.
3. Studies published in any language, between January 2010 and September 2020.
4. Studies in which the samples were characterised by full-term gestations (between 37 and 42 weeks of gestation), with a single foetus, and breech presentation.

The authors decided to establish observational studies as an inclusion criterion as a review restricted to randomised controlled trials would have given an incomplete summary of the effects of a treatment, due to potential harms. Therefore, ClinicalTrials.gov was not consulted. The studies published before 2010 were excluded because recent scientific publications have turned other previously published ones into outdated evidence, and the aimed was to gather the latest reliable results. In addition, studies where foetuses had lethal congenital abnormalities and caesareans made by other obstetric indications such as multiple pregnancy or intrauterine foetal deaths were also excluded.

The selection of studies was carried out in three stages. First, after reviewing the titles, all relevant literature was retrieved from the respective databases. Second, summaries of all recovered articles were reviewed and then grouped as “eligible for inclusion” or “Not eligible for inclusion”. Third, articles that were grouped as “eligible for inclusion” were revised in detail for the final decision.

The entire process of selection, the quality assessment and also data extraction were carried out by two investigators independently. Each study was individually evaluated by one of the researchers and results were shared. In case of discrepancies, both researchers discussed their arguments and agreement was reached by consensus; occasionally, a third researcher’s assessment was required.

### Methodological quality of the included studies

The methodological quality of the eligible articles was assessed according to the Newcastle-Ottawa scale. This scale was designed for assessing the quality of non-randomised studies included in a systematic review and/or meta-analyses. It contains eight items organised in three dimensions: the selection of the study groups (four items); the comparability of the groups (one item); and the ascertainment of the outcome (three items). Studies were evaluated following a star system such that each item can be awarded a maximum of one star, excepting the item related to comparability, which allows the assignment of two stars. The total score ranges between zero and nine stars [15,16].

### Data extraction

To structure the collected data, all results compatible with perinatal mortality and morbidity in relation to the type of delivery with breech presentation in full-term gestations (between 37 and 42 weeks of gestation) with a single foetus were searched internationally. The results of the different items were compared on the basis of the primary outcomes, which were neonatal mortality, perinatal trauma, Apgar, neonatal ICU admittance, and maternal morbidity.

Data were extracted using a standard Excel (Microsoft, Redmond, WA, USA) spreadsheet. The extracted data included: the name of the first author, year of publication, period of study, country where the study was conducted in, conclusion of the study, sample size, type of delivery, intrapartum and neonatal mortality, perinatal trauma, Apgar score at the first and fifth minute of life, neonatal ICU admissions, and severe maternal morbidity.

In this review, neonatal mortality was considered as deaths before 7 days of age after birth. The WHO establishes early neonatal mortality up to the seventh day of life. Complications at birth as a result of childbirth are manifested in the first 7 days [17]. In fact, all the observational studies included in the present meta-analysis took this same period of time as a reference. Perinatal trauma included collarbone fracture, humerus or femur, intracerebral bleeding, cephalic haematoma, facial paralysis, brachial plexus injury, and other trauma.

For this study, severe maternal morbidity was considered as unexpected labour and delivery outcomes that result in significant short-term or long-term consequences for the woman's health. Serious complications of the intervention, whether caesarean or delivery, severe postpartum haemorrhages, neurological problems, sepsis, lung, kidney, or cardiac problems were included [18].

### Statistical analysis

A meta-analysis was performed to evaluate each of the indicators that could measure morbidity and mortality in planned vaginal delivery and scheduled caesarean for breech presentations for both the newborn and the mother.

The Mantel-Haenszel method was used to obtain typical RR estimates and 95% confidence intervals (CI). Heterogeneity was determined using the Cochran’s Q χ^2^ test and the I^2^ values for the following variables:

(1) Early and incipient neonatal death, (2) birth trauma, (3) Apgar test score at 5 minutes, (4) neonatal admission to ICU, (5) severe maternal morbidity.

Heterogeneity between studies was assessed by calculating values for I^2^ and *P* values. Due to the high I^2^, an important statistic for assessing heterogeneity, the random effects method was used. The I^2^ value was interpreted as without heterogeneity (0%), low heterogeneity (<40%), moderate heterogeneity (<60%), substantial heterogeneity (<75%) and considerable heterogeneity (≥75%) [19]. The stability of the overall RR in the withdrawal of any of the studies was performed by sensitivity analysis (treating one study at a time). All meta-analyses were performed using the Epidat Software 3.0 (Xunta de Galicia, Santiago de Compostela, Spain).

## RESULTS

### Description of the included studies

**Figure 1:**
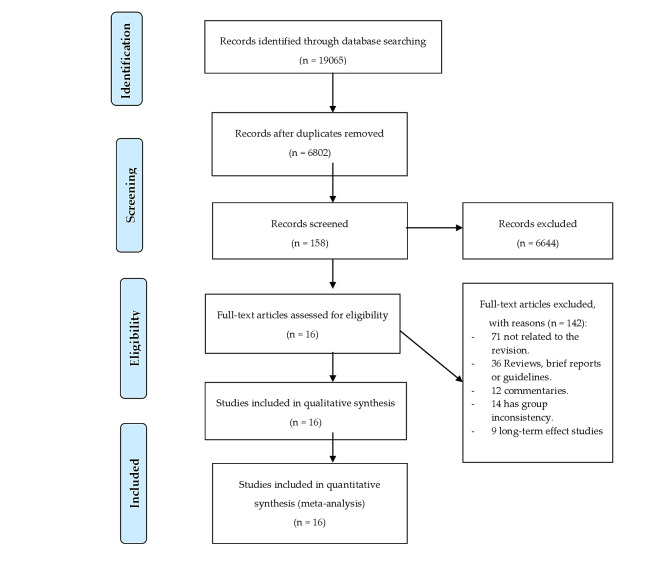
PRISMA flowchart.

The initial electronic search yielded a total of 19 055 references, and after removing duplicate records, 6802 references were reviewed. Of these, after reading the title and abstract, 6644 references were deleted for not meeting the inclusion criteria, so 158 were selected for full text review. Following the research protocol, 142 were excluded because they were not related to the current revision, because some made comparisons between breech and vertex presentation, and others assessed long-term maternal and neonatal complications. Finally, 16 articles were selected for meta-analysis [10,20-34]. The selection process is shown in **Figure 1**.

Of the 16 studies, 10 had been conducted in Europe, 2 in Asia, 2 in Oceania, and 2 in Africa. Of these, 4 were in favour of elective caesarean to minimise neonatal morbidity but recognised that this increased long-term maternal morbidity by conditioning the type of birth for a future pregnancy [10,21,27,34]. Two of the reviewed studies found that caesarean reduced the risk of neonatal mortality [10,21]. However, 12 of the studies involved in the meta-analysis concluded that vaginal delivery could be an acceptable option in breech presentation provided that strict criteria for the selection of cases were established [20,22-26,28-33]. Sample sizes for the studies included ranged from 111 to 58 320 (**Table 1**).

**Table 1:** Characteristics of the studies included in the meta-analysis

Author, year	Country	Study period	Type of study	Number of breech deliveries	Attempted deliveries	Planned caesareans	Quality assessment	Conclusions
Vlemmix F et al, 2014 [9]	Netherlands	1999-2007	Cohort	58 320	27 817	30 503	High quality	The more caesareans, the less mortality and neonatal morbidity, but mothers will end up with scarred uterus for future pregnancies.
Babovic I et al, 2010 [20]	Serbia	2007-2008	Cohort	401	267	134	High quality	No deaths or nervous system injuries in any of the groups
Tharin HJE et al, 2011 [21]	Denmark	Jan1997-2008	Cohort	21 803	7039	14 764	High quality	Caesarean decreases child mortality, Apgar values at 5 min are higher, and less ICU admittances are required. Strict selection does not reduce the risks of vaginal delivery.
Singh A et al, 2012 [22]	India	2007-2009	Cohort	111	60	51	High quality	With strict selection of women, vaginal delivery would be a feasible option.
Toivonen E et al, 2012 [23]	Finlandia	2004-2009	Cohort	751	254	497	High quality	Vaginal delivery would be an acceptable option by accurately selecting cases.
Vistad I et al, 2013 [24]	Norway	2001-2011	Cohort	568	289	279	High quality	Vaginal delivery would be an acceptable option through strict selection and control.
Foster BA et al 2014 [25]	Australia	Oct 1999- Dec 2010	Cohort	766	243	523	High quality	Fewer complications in vaginal delivery than in studies in other countries. Therefore, an acceptable option.
Babovic I et al, 2016 [26]	Serbia	2013	Cohort	146	72	74	High quality	Caesarean delivery is best in nulliparous women over 35 y of age or women whose estimated foetal weight is greater than 3500 g. For all other cases, vaginal delivery would be an acceptable option.
Bin YS et al, 2016 [27]	Australia	2009-2012	Cohort	5197	352	4845	High quality	Vaginal delivery causes more neonatal and maternal morbidity. Therefore, caesarean is recommended for all breech births.
Högberg U et al, 2016 [28]	Tanzania	1999-2010	Cohort	1655	908	747	High quality	Low-income country. Vaginal delivery is associated with perinatal risk. But caesarean doesn't work better. Therefore, they opt for vaginal delivery.
Abdessalami S et al, 2017 [29]	Netherlands	2007-2015	Cohort	309	119	190	High quality	Vaginal delivery is an option in selected women with low risk but is strongly influenced by the counselling technique, which is not always based on scientific evidence.
Fonseca A et al, 2017 [30]	Portugal	Jan 2012 – Oct 2014	Cohort	1327	65	1262	High quality	Both delivery pathways are acceptable, with no more risks in vaginal deliveries than in caesareans.
Debero-Mere T et al, 2017 [31]	Ethiopia	2013-2016	Cohort	384	317	67	High quality	Low-income country. Vaginal delivery implies a higher risk in women over the age of 35, with large foetuses and poor cervical conditions. If cases are accurately selected, vaginal delivery seems a safe option.
Louwen F et al, 2017 [32]	Germany	Jan 204- Jun 2011	Cohort	747	433	314	High quality	Vaginal delivery with the woman upright is more successful than in the dorsal position. Therefore, vaginal delivery is an acceptable option.
Grupta V et al, 2019 [33]	India	2016-2017	Cohort	180	127	53	High quality	Vaginal delivery is an option when carefully selecting cases.
Vinkenvleugel DAM et al 2020 [34]	Netherlands	2011-2017	Cohort	1620	425	1195	High quality	Elective caesarean would be safer for newborns, but worse for the mother as it conditions the birth pathway for future pregnancy. Therefore, vaginal delivery will be attempted as long as it can be cared for by an experienced person and the conditions are strictly selected.

Regarding methodological quality assessment, the included studies were scored from 5 to 9 stars according to de Newcastle-Ottawa scale (**Table 2**). The publication bias was analysed, and results were summarised in Figure S1 and Figure S2 in the **Online Supplementary Document**.

**Table 2:** Methodological quality assessment and quality of evidence*

Author, year	Selection	Comparability	Outcome	GRADE (quality of evidence)
Abdessalami S et al, 2017 [29]	* * * *	* *	* * *	2++
Babovic I et al, 2010 [20]	* * * *	* *	* * *	2++
Babovic I et al, 2016 [26]	* * * *	* *	* * *	2++
Bin YS et al, 2016 [27]	* * * *	* *	* * *	2++
Debero-Mere T et al, 2017 [31]	* * *	* *	* *	2++
Fonseca A et al, 2017 [30]	* * * *	* *	* * *	2++
Foster Ab et al 2014 [25]	* * * *	* *	* * *	2++
Grupta V et al, 2019 [33]	* * * *	* *	* * *	2++
Högberg U et al, 2016 [28]	* * * *	* *	* * *	2++
Louwen F et al, 2017 [32]	* * * *	* *	* * *	2++
Singh A et al, 2012 [22]	* * *	* *	* * *	2+
Tharin HJE et al, 2011 [21]	* *	*	* *	2
Toivonen E et al, 2012 [23]	* * * *	-	* * *	2
Vinkenvleugel DAM et al 2020 [34]	* * * *	* *	* * *	2++
Vistad I et al, 2013 [24]	* * * *	* *	* * *	2++
Vlemmix F et al, 2014 [9]	* * *	*	* * *	2+

*Selection: maximum score ****, Comparability: maximum score **, Outcome: maximum score ***. GRADE: 1 = high, 2 = moderate, 3 = low, 4 = very low.

### Findings of the meta-analysis

**Figure 2:**
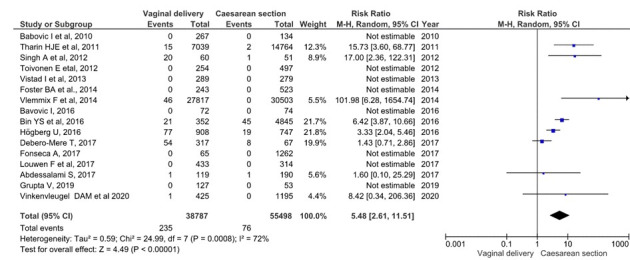
Meta-analysis of perinatal deaths in full-term singleton breech presentation (planned vaginal delivery vs planned caesarean section) (n = 94 285).

Perinatal mortality analysis consisted of 16 studies and included 94 285 single foetus, full-term, breech presentation deliveries (38 787 planned vaginal deliveries and 55 498 scheduled caesareans). As shown in **Figure 2**, perinatal mortality (intrapartum and early neonatal death) in the planned vaginal delivery group was 235 (0.6%), and in the elective caesarean group it was 76 (0.14%) (10,20-34). The grouped meta-analysis has shown that the risk of perinatal mortality was 5.48 (95% CI = 2.61 to 11.51) times higher in the vaginal delivery group than in the planned caesarean group. The overall heterogeneity of the tests showed substantial variability between studies (I^2^ = 72%). Sensitivity analysis showed that the overall RR was 3.10; 95% CI = 1.8 - 5.2 (the detailed sensitivity analysis of each variable are summarised in Table S2 in the **Online Supplementary Document**).

**Figure 3:**
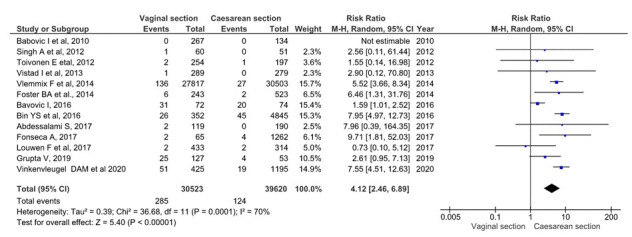
Meta-analysis of perinatal trauma in term singleton breech presentation (planned vaginal delivery vs planned caesarean section) (n = 70 143).

Perinatal trauma analysis included 70 143 single foetus, full-term, breech presentation deliveries (30 523 planned vaginal deliveries and 39 620 planned caesareans). As shown in **Figure 3**, perinatal trauma in the planned vaginal delivery group was 285 (0.41%), and in the elective caesarean group it was 124 (0.18%) [10,20,22-25,27,29,30,32-34]. The grouped meta-analysis showed a 4.12 (95% CI = 2.46 to 6.89) times increased risk of birth trauma in the planned vaginal delivery group. The overall heterogeneity of the tests showed substantial variability between studies (I^2^ = 70%). The sensitivity analysis showed that the overall RR was 3.6 95% CI = 2.17-6.09.

**Figure 4:**
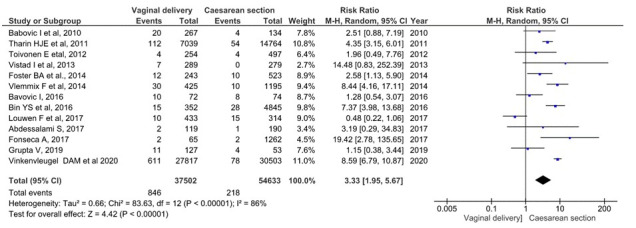
Meta-analysis of 5-minute Apgar <7 score in term singleton breech presentation (planned vaginal delivery vs planned caesarean section) (n = 92 135).

Regarding the Apgar score at minute 5, 13 studies were assessed including 92 135 deliveries with breech, single foetus, and term presentations (37 502 planned vaginal deliveries and 54 633 planned caesareans). 846 (0.92%) neonates of the planned vaginal delivery group had an Apgar below 7 points at the 5th minute of life. Also, in the planned caesarean group, there were 218 (0.24%) neonates whose test score was less than 7 points at 5 minutes of life [10,20,21,23-27,29,30,32,33] (**Figure 4**). The grouped meta-analysis showed a nearly 3.33 (95% CI = 1.95-5.67) times higher risk of the Apgar test having a score of less than 7 points in the planned vaginal delivery group. The overall heterogeneity of the tests showed considerable variability between studies (I^2^ = 86%). However, the sensitivity analysis showed that the overall RR was 3.8 95% CI = 2.07-7.25.

**Figure 5:**
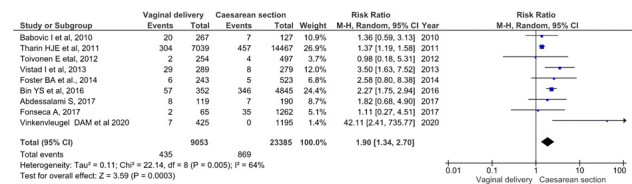
Meta-analysis of intensive care unit (ICU) admissions in term, singleton breech presentation (planned vaginal delivery vs planned caesarean section) (n = 32 438).

Admittance to neonatal ICU assessment included 9 studies, 32 438 single foetus, full-term, breech presentation deliveries (9053 planned vaginal deliveries and 23 385 elective caesareans) were included. In the planned vaginal delivery group, there were 435 (1.86%) admittances at the ICU of newborns, while in the planned caesarean group, the figure was 869 (3.72%) [20,21,23-25,27,29,30] (**Figure 5**). The grouped meta-analysis showed a 1.90 (95% CI = 1.34-2.70) times increased risk of admittance to ICU in the planned vaginal delivery group. The overall heterogeneity of the tests showed substantial variability between studies (I^2^ = 64%). However, the sensitivity analysis showed that the overall RR was 1.9 (95% CI = 1.36-2.76).

**Figure 6:**
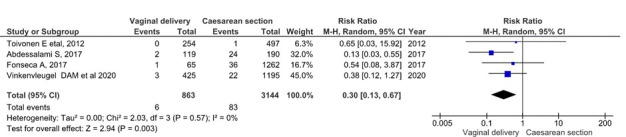
Meta-analysis of severe maternal morbidity in term singleton breech presentation (planned vaginal delivery vs planned caesarean section) (n = 4007).

Regarding maternal morbidity, the analysis included 4 studies. 4007 single foetus, full-term, breech presentation deliveries were included (863 planned vaginal deliveries and 3144 planned caesareans) [23,27,30,34] (**Figure 6**). Maternal morbidity was found in 6 cases (0.69%) for the planned vaginal delivery group, and in 83 cases (2.64%) for the planned caesarean group. The grouped meta-analysis showed a 0.30 (95% CI = 0.13-0.67) times reduced risk of severe maternal morbidity in the planned vaginal delivery group than in the planned caesarean group. The overall heterogeneity of the tests showed very low variability between studies (I^2^ = 0%).

## DISCUSSION

### Main findings

The meta-analysis has shown a decreased relative risk perinatal mortality and morbidity in a planned caesarean as compared with a vaginal delivery when breech presentation.

### Interpretation

Regardless of whether childbirth is done vaginally or through caesarean delivery, morbidity and mortality rates have been represented higher at breech births than at cephalic births [35]. Since the publication of TBT [3], several studies have shown increased morbidity and perinatal mortality with breech presentations in planned vaginal delivery vs planned caesarean [9,21,36,37]. These results were consistent with TBT [3] and PREMODA results [5].

Although the potential biases associated with the observational design of the studies included in this meta-analysis must be recognised, with the consequent caution in comparing results with similar previous studies, our results were in line with previous meta-analyses. According to Berhan et al. [12], the relative risk of perinatal mortality, trauma at birth, and Apgar at the fifth minute of life were higher in the planned vaginal delivery than in planned caesarean for term singleton breech (3.4 vs 6.3; 3.1 vs 4.2; and 4.7 vs 2.99, respectively). Our study, despite having included only observational studies, agreed with these outcomes.

For the severe maternal morbidity indicator, the present meta-analysis showed a relative risk of 0.30 in favour of vaginal delivery. This means that vaginal delivery is a protective factor against severe maternal morbidity. Although the risk is low, maternal morbidity and mortality increase as a result of complications of a planned caesarean for breech presentations [21,36]. Several studies claimed that planned caesarean may increase the risks for the mother as a result of scarred uterus [9,21,34], so the relative safety of planned caesarean should be weighed [9,38].

In the absence of a contraindication for vaginal delivery, a woman with a breech presentation foetus must be truthfully informed, considering the scientific evidence so far, of the risks and benefits of vaginal breech delivery and elective caesarean, so that the woman can decide and consent to the desired type of delivery [29,39]. The woman's decision must be respected and, to do so, the staff attending births must be trained and updated in the assistance of breech vaginal deliveries [39,40]. Otherwise, the woman will be denied a medical treatment option to which she could have turned to [40].

Regardless of the way of planning the type of delivery, vaginal delivery in breech presentation will always exist, as a delivery may always become urgent and present with these characteristics. Therefore, it is essential that staff attending births do not lose this ability and master it in order to provide quality health care to women [39].

### Strengths and limitations

The risks for neonatal mortality and maternal morbidity implies an ethical dilemma: assuming either the risk of neonatal mortality or the risk of severe maternal morbidity. The risk of neonatal mortality was higher; therefore, we would only consider exposing the mother and foetus to vaginal delivery in the case of good obstetric conditions and given that the health care professional is well trained and experienced in these procedures. Otherwise, we recommend delivery by caesarean section. Our study bases the practice of individualisation on decision-making when choosing the type of delivery in unique gestations with full-term foetuses and breech presentation. Each pregnancy should assess the risks individually, considering the woman's preferences and the context, and seeking a balance between neonatal mortality and maternal morbidity.

Some limitations have been found in conducting this research, starting with the great variability regarding the size of the samples. Studies with very small samples have had little weight when calculating RR in the grouped meta-analysis, while studies with a very large sample size had much more weight. For this reason, we have had to accept a relatively high (moderate) percentage of heterogeneity (I^2^) in some meta-analyses as, if eliminated, the sample would be drastically reduced.

Vaginal, breech, full-term delivery with a single foetus had a higher risk of morbidity and perinatal mortality than caesarean delivery under the same conditions. Still, the results of this meta-analysis suggested that the risk of vaginal breech delivery is lower than in the results of other previously published studies [29-31,33,34].

Additionally, the potential bias accompanying observational studies should be acknowledged, given the Newcastle-Ottawa tool identified some items with lack of quality. Therefore, caution is suggested when comparing and generalising the results.

## CONCLUSIONS

Term breech birth risks have been analysed according to two possibilities: Vaginal delivery and caesarean delivery risks. Caesarean had high rates of postpartum maternal morbidity. Also, there is no evidence of reduced child perinatal morbidity or mortality. Otherwise, there is no contraindication for vaginal delivery in breech presentation in selected pregnant women and in the presence of experienced health workers.

Our results could help in decision-making related to breech delivery, individualising the decision for each case by knowing the risks associated with each option. From an ethical perspective, the issue addressed in the review is highly sensitive, considering the risk of maternal morbidity and the risk of neonatal mortality. For this reason, further research is suggested that consolidates the available evidence for decision-making between the studied delivery methods.

## Additional material


Online Supplementary Document
