# Supplementary material for: Maternal and fetal risks of planned vaginal breech delivery vs planned caesarean section for term breech birth: A systematic review and meta-analysis
Source: J Glob Health. 2022 Jul 16;12:04055. doi: 10.7189/jogh.12.04055 (PMC9284475; doi:10.7189/jogh.12.04055)
Supplement: Online Supplementary Document [file jogh-12-04055-s001.pdf]

Table S1. Search strategies.

Databases	Search date	Search strategy	Number of results	Selected results
Cochrane	10/09/2020	#1 (Breech presentation) (word variations have been searched)<br>#2 (Breech Birth) (word variations have been searched)<br>#3 (Breech Delivery) (word variations have been searched)	199<br>128<br>199	2<br>1<br>1
Medline	11/09/2020	Breech [Mesh] AND presentation [Mesh].<br>Breech [Mesh] AND birth[Mesh]<br>Breech [Mesh] AND delivery [Mesh]	1125<br>757<br>1107	20<br>24<br>8
Scopus	15/09/2020	(“breech” AND “presentation”)<br>(“breech” AND “birth”)<br>(“breech” AND “delivery”)	2175<br>1257<br>1575	16<br>12<br>8
Embase	17/09/2020	('breech presentation'/exp OR 'breech presentation') AND ('birth'/exp OR birth)<br>('breech presentation'/exp OR 'breech presentation') AND ('obstetric delivery'/exp OR 'obstetric delivery')	2313<br>4196	28<br>20
Web of Science	20/09/2020	#1 TEMA: (breech presentation) Bases de datos= WOS, CCC, DIIDW, KJD, MEDLINE, RSCI, SCIELO Período de tiempo=2010-2020<br>#2 TEMA: (breech birth) Bases de datos= WOS, CCC, DIIDW, KJD, MEDLINE, RSCI, SCIELO Período de tiempo=2010-2020<br>#3 TEMA: (breech delivery) Bases de datos= WOS, CCC, DIIDW, KJD, MEDLINE, RSCI, SCIELO Período de tiempo=2010-2020	1531<br>1103<br>1379	7<br>5<br>6
Cuiden	21/09/2020	("breech")AND("presentation")<br>("breech")AND("birth")<br>("breech")AND("delivery")	13<br>3<br>5	0<br><br>0
TOTAL			19065	158

Table S2. Sensitivity analysis of perinatal mortality, perinatal trauma Apgar score at 5 min and admittance to neonatal ICU.

**Sensitivity analysis of perinatal mortality**					
Study	Year	*N*	RR	CI 95%	
				Inferior	Superior
Babovic et al [20]	2010	93540	3.2833	1.9285	5.5897
Tharin et al [21]	2011	72140	2.6862	1.6265	4.4363
Singh et al [22]	2012	93832	2.8170	1.6748	4.7381
Toivonen et al [23]	2012	93190	3.1572	1.8264	5.4576
Vistad et al [24]	2013	93373	3.2261	1.8763	5.5472
Vlemmix et al [9]	2014	35622	2.6615	1.7052	4.1538
Foster et al [25]	2014	93175	3.1475	1.8202	5.4427
Bavovic et al [26]	2016	93795	3.2213	1.8721	5.5429
Bin et al [27]	2016	89106	3.2355	1.6652	6.2868
Högberg et al [28]	2016	92288	3.0951	1.6108	5.9471
Abdessalami et al [29]	2017	93634	3.1776	1.8400	5.4877
Fonseca et al [30]	2017	92614	2.9254	1.7252	4.9604
Debero-Mere et al [31]	2017	93559	3.5659	2.0195	6.2967
Louwen et al [32]	2017	93194	3.2521	1.8984	5.5709
Grupta et al [33]	2019	93761	3.2982	1.9445	5.5942
Vinkenvleugel et al [34]	2020	92322	3.1199	1.8036	5.3969
GLOBAL		93943	3.1020	1.8316	5.2536
**Sensitivity analysis of perinatal trauma**					
Babovic et al [20]	2010	70045	3.8718	2.3108	6.4871
Singh et al [22]	2012	70336	3.8015	2.2541	6.4110
Toivonen et al [23]	2012	69697	3.6137	2.1200	6.1599
Vistad et al [24]	2013	69879	3.7849	2.2418	6.3900
Vlemmix et al [9]	2014	12128	3.2750	1.7601	6.0939
Foster et al [25]	2014	69682	3.4801	2.0229	5.9871
Bavovic et al [26]	2016	70302	5.0013	3.4041	7.3481
Bin et al [27]	2016	65251	3.1925	1.8218	5.5944
Abdessalami et al [29]	2017	70138	3.6419	2.1371	6.2051
Fonseca et al [30]	2017	69121	3.4099	1.9935	5.8327
Louwen et al [32]	2017	69701	3.9741	2.3698	6.6645
Grupta et al [33]	2019	70268	3.7509	2.1523	6.5369
Vinkenvleugel et al [34]	2020	68828	3.2096	1.8109	5.6885
GLOBAL		70448	3.6415	2.1747	6.0979
**Sensitivity analysis of the APGAR score at 5 min.**					
Babovic et al [20]	2010	91379	4.0294	2.0857	7.7843
Tharin et al [21]	2011	69977	3.8313	1.7663	8.3106
Toivonen et al [23]	2012	91029	4.0814	2.1297	7.8217
Vistad et al [24]	2013	91211	3.7754	1.9792	7.2014
Vlemmix et al [9]	2014	33460	3.5707	1.6588	7.6863
Foster et al [25]	2014	91014	4.0329	2.0749	7.8388
Bavovic et al [26]	2016	91634	4.3028	2.2644	8.1763
Bin et al [27]	2016	86583	3.6386	1.8265	7.2485

Abdessalami et al [29]	2017	91471	3.9153	2.0596	7.4429
Fonseca et al [30]	2017	90453	3.5598	1.8697	6.7779
Louwen et al [32]	2017	91033	4.7856	2.7101	8.4506
Grupta et al [33]	2019	91600	4.2995	2.2622	8.1716
Vinkenvleugel et al [34]	2020	90516	2.9922	1.6967	5.2769
GLOBAL		91780	3.8832	2.0778	7.2574
**Sensitivity analysis of admittance to neonatal ICU**					
Babovic et al [20]	2010	32045	2.0318	1.3768	.9983
Tharin et al [21]	2011	10940	2.2200	1.5988	3.0827
Toivonen et al [23]	2012	31695	2.0041	1.3897	2.8902
Vistad et al [24]	2013	31878	1.7870	1.2519	2.5508
Foster et al [25]	2014	31680	1.9087	1.3166	2.7672
Bin et al [27]	2016	27249	1.8780	1.2251	2.8788
Abdessalami et al [29]	2017	32137	1.9661	1.3420	2.8806
Fonseca et al [30]	2017	31119	2.0127	1.3899	2.9145
Vinkenvleugel et al [34]	2020	30825	1.8212	1.3250	2.5034
GLOBAL		32446	1.8446	1.3675	2.7653

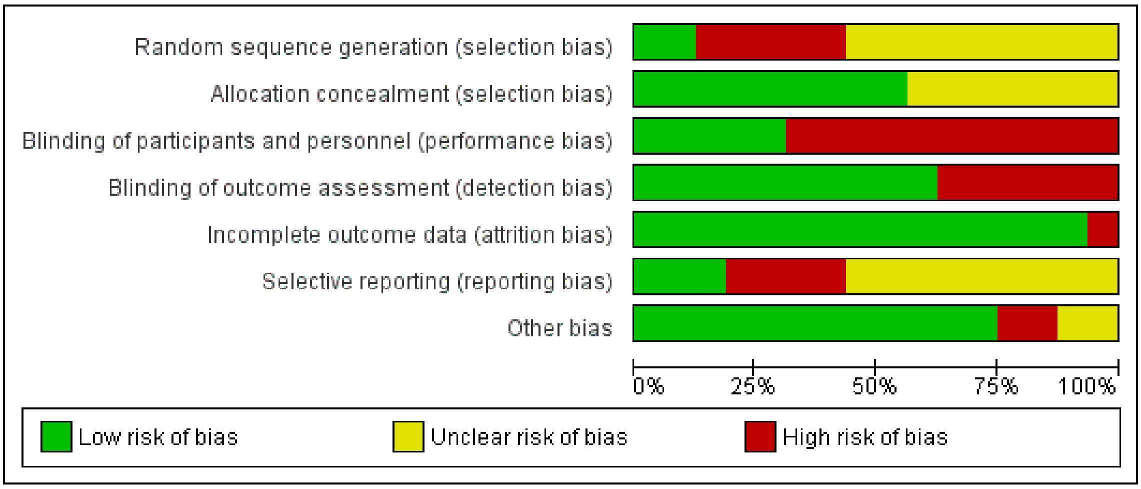

Figure S1. Publication bias analysis.

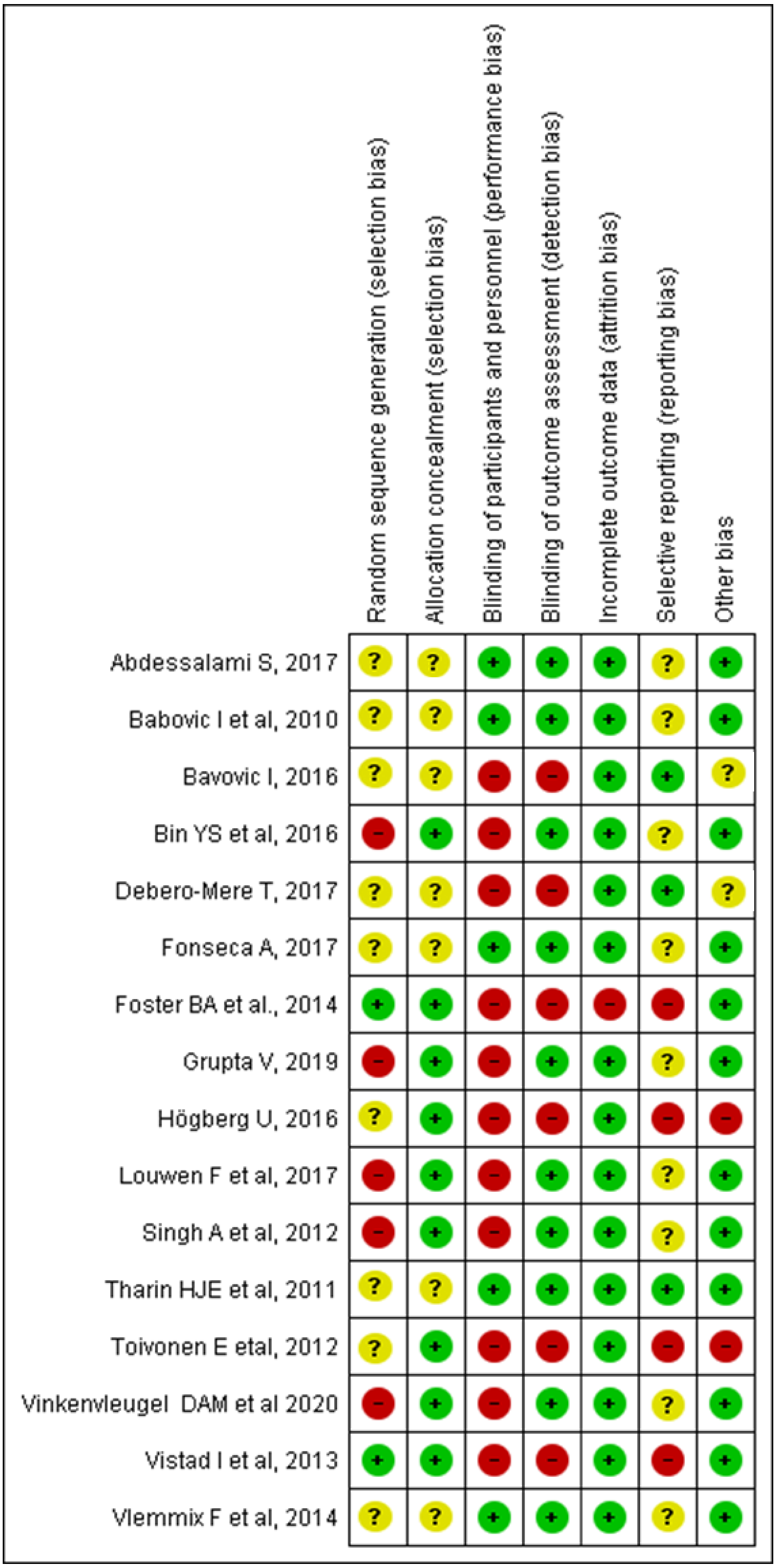

Figure S2. Summary of publication bias of the included studies.
